# Supplementary material for: Light microscopy based approach for mapping connectivity with molecular specificity
Source: Nat Commun. 2020 Sep 15;11:4632. doi: 10.1038/s41467-020-18422-8 (PMC7493953; doi:10.1038/s41467-020-18422-8)
Supplement: Supplementary file 1 — Supplementary Information [file 41467_2020_18422_MOESM1_ESM.pdf]

Title: Light microscopy based approach for mapping connectivity with molecular specificity

Shen et al.

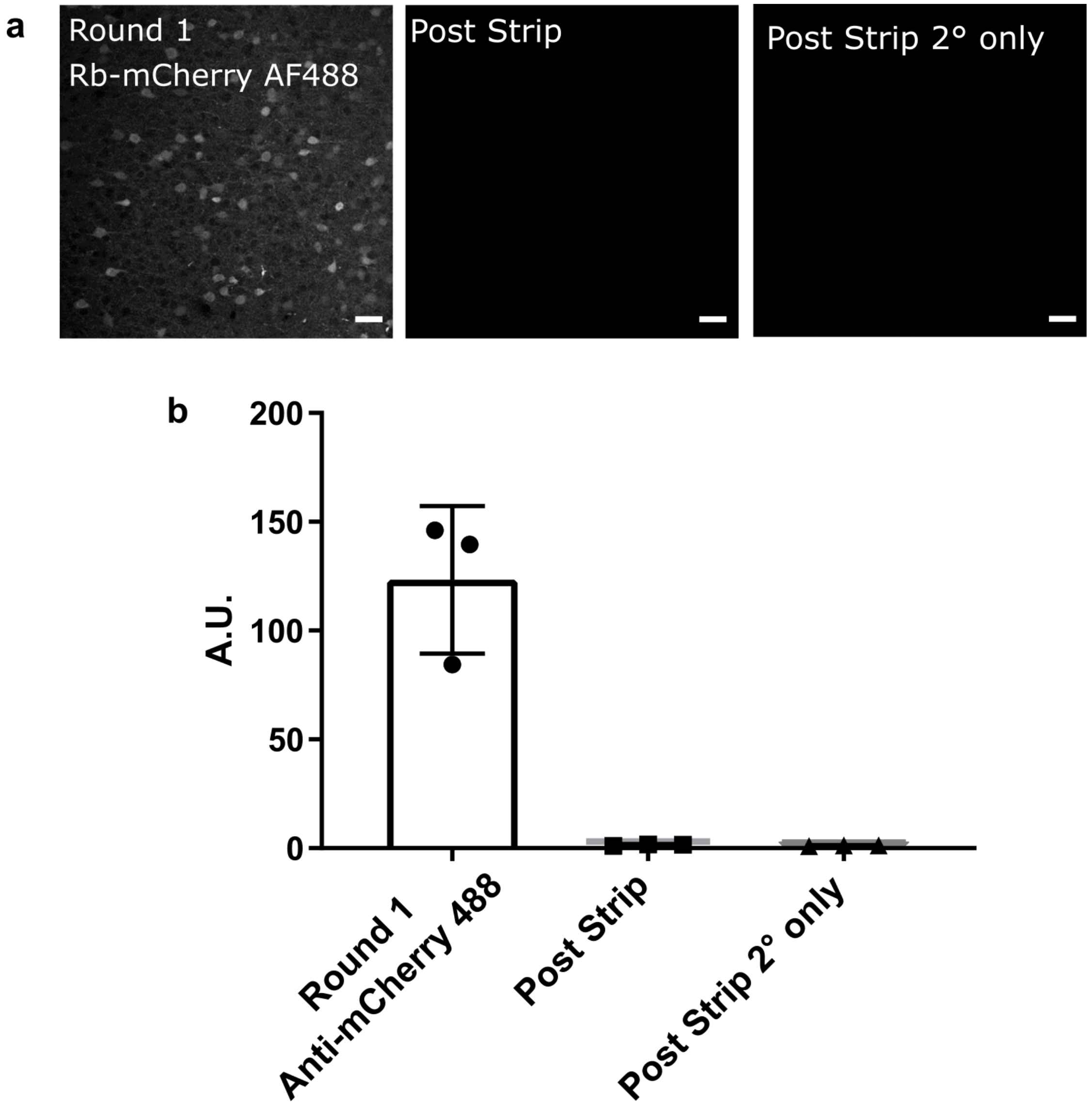


**Supplementary Figure 1. Heat and SDS are efficient at eluting antibody from miriEx gel-tissue hybrids.** (a) VGAT-Cre x Ai14 tissue sections were processed with miriEx and TdTomato+ neurons were immunostained with rb-mCherry antibody. The samples were then stripped using heat/SDS and re-imaged (see “Antibody elution” section in methods). The samples were then stained with only secondary antibody to check that the primary antibody was stripped off. (b) Quantification of fluorescence intensity seen in a (n=3 separate samples from 1 animal). Error bars: standard deviation. Scale bars: (a) 50 μm (pre-expansion size). Expansion factor: (a) ~2x. See Supplementary Table 1 for more details.

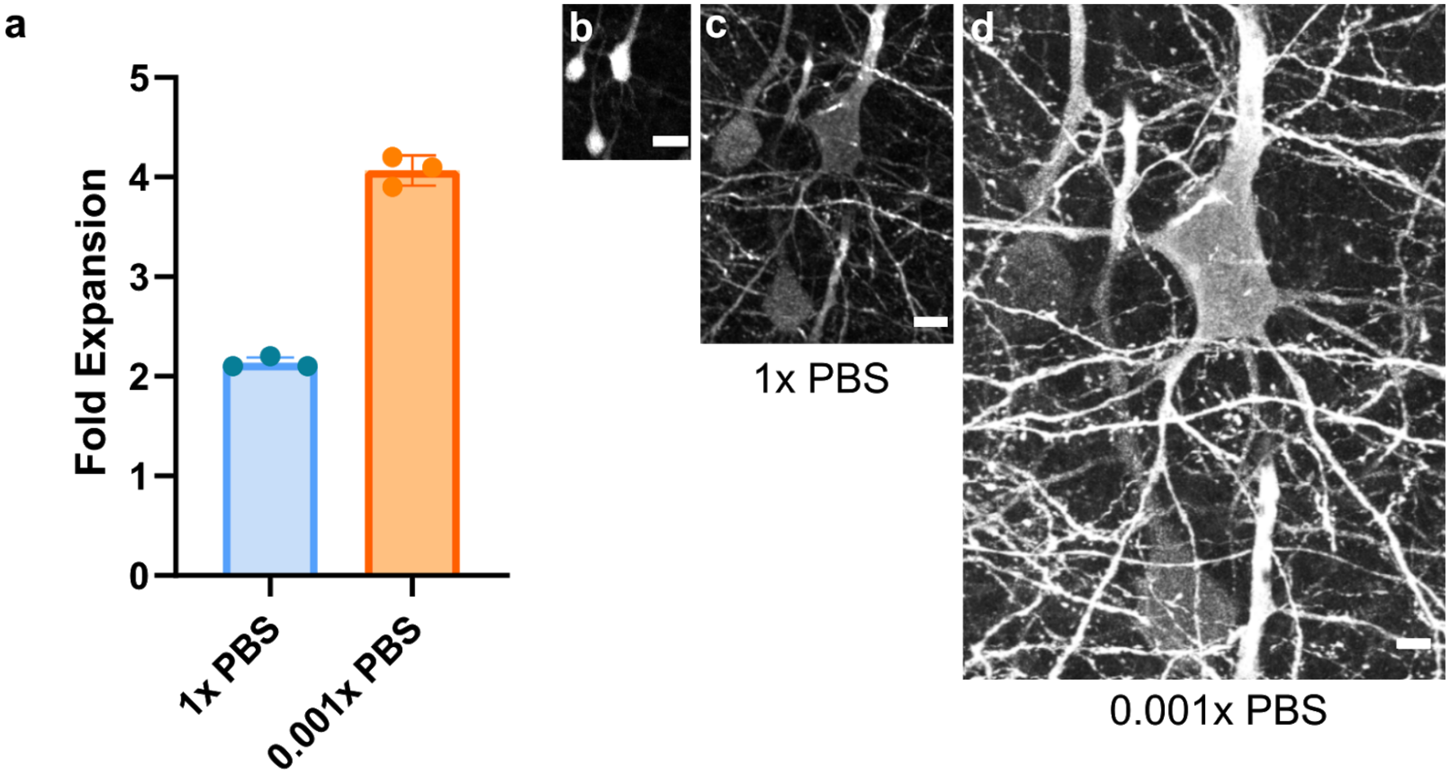


**Supplementary Figure 2. miriEx gel-tissue hybrid expansion.** (a) miriEx gels expand approximately ~2 fold in 1xPBS, and ~4 fold in 0.001xPBS (n=3 samples). (b-d) Same field of view from Thy1-YFP-H layer 5 somatosensory cortex imaged in three conditions: before miriEx processing, after miriEx processing in 1x PBS, and after miriEx processing in 0.001x PBS respectively. Error bars: Standard Deviation. Scale bars: (b-d) 20 µm. See Supplementary Table 1 for more details.

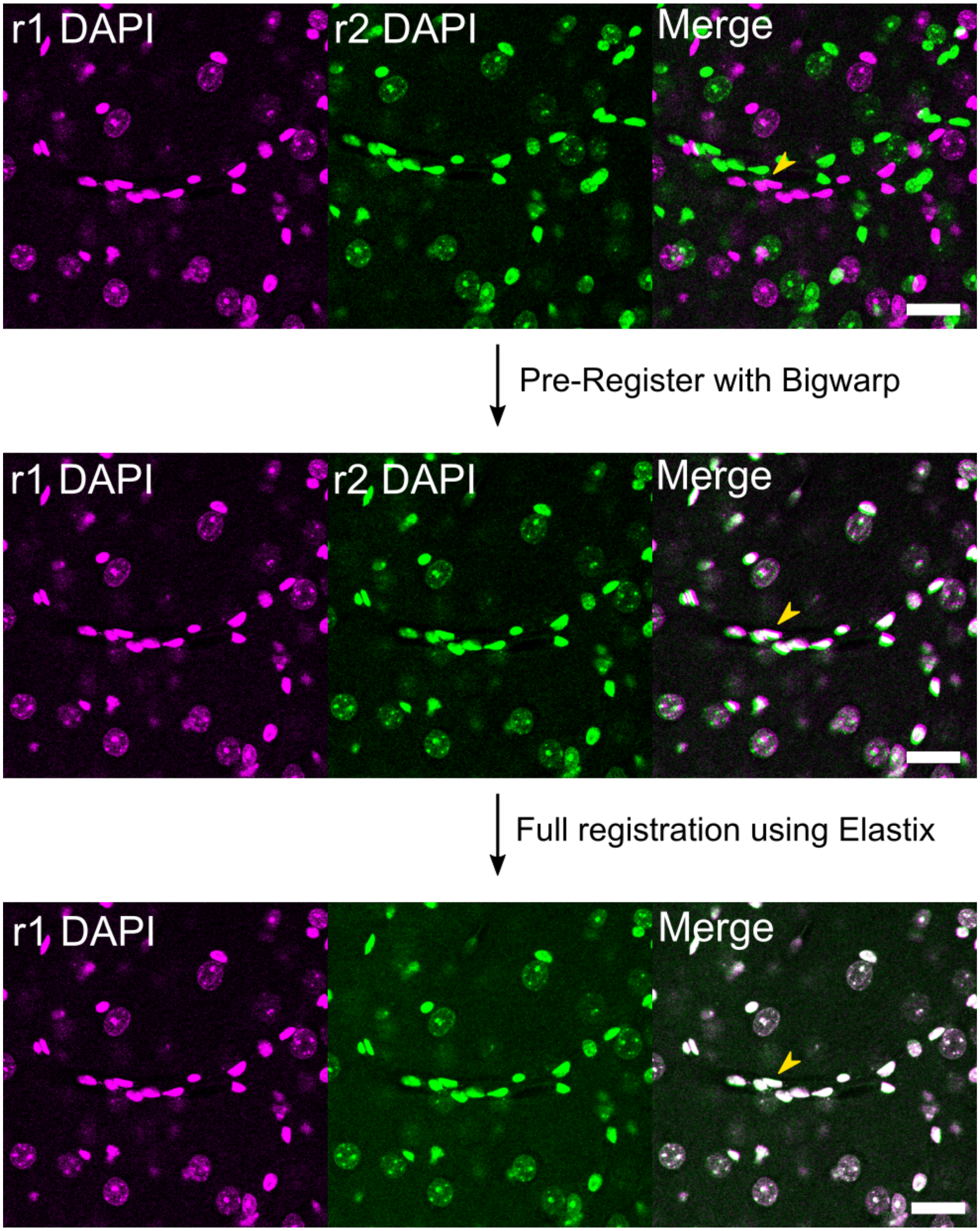


**Supplementary Figure 3. Image registration pipeline between different rounds of miriEx.** Example of how DAPI nuclear stain can be used as a fiduciary channel for registration. First, different rounds of DAPI are pre-aligned manually using Bigwarp plugin from Fiji/ImageJ. Then, the two rounds are automatically aligned using Elastix by using a non-linear B-spline grid. We found that a coarse pre-alignment step improved the Elastix registration. The resulting transformation was then applied to all the other antibody channels from round 2 to register them to round 1. Scale bars: 25 μm (pre-expansion size).

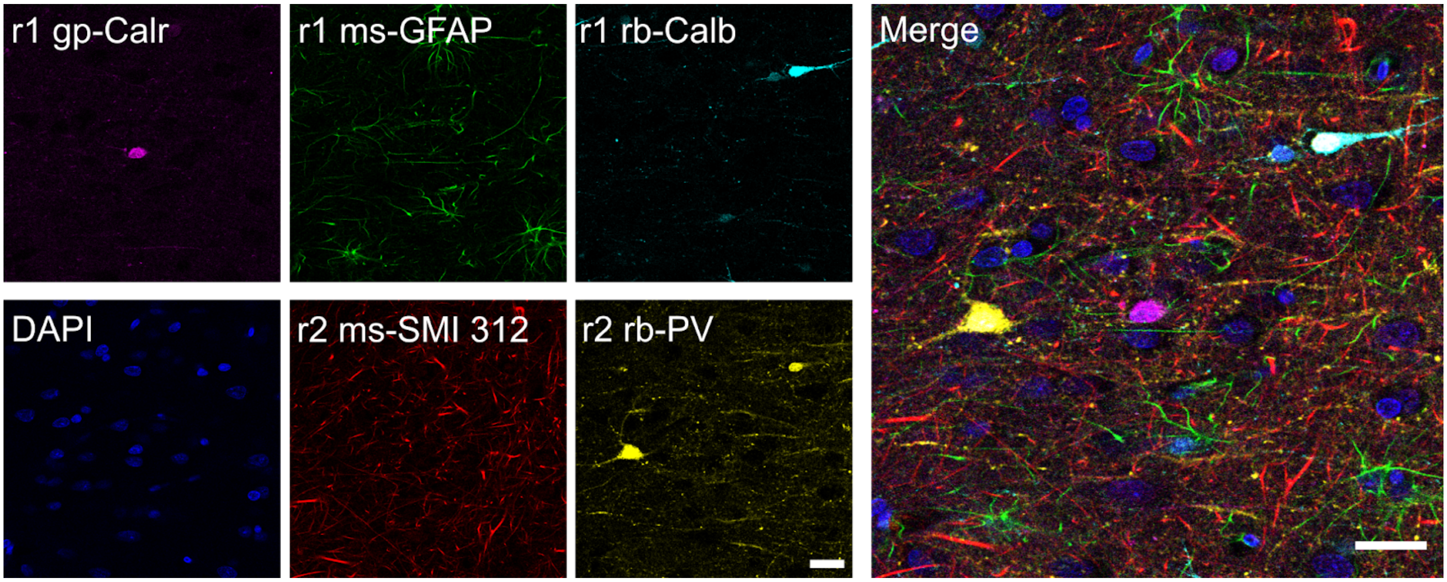


**Supplementary Figure 4. miriEx enables multiplexed immunostaining in formalin fixed human brain tissue.** 100 µm formalin fixed human brain tissue was processed with miriEx to label 6 different targets across 2 rounds of immunostaining. DAPI was stained in each round to use as a fiduciary channel for registration. Calr, Calretinin; PV, parvalbumin; Calb, calbindin; GFAP, glial fibrillary acidic protein. Scale bars: 25 µm (pre-expansion size). Expansion factor: ~2x. See Supplementary Table 1 for more details.

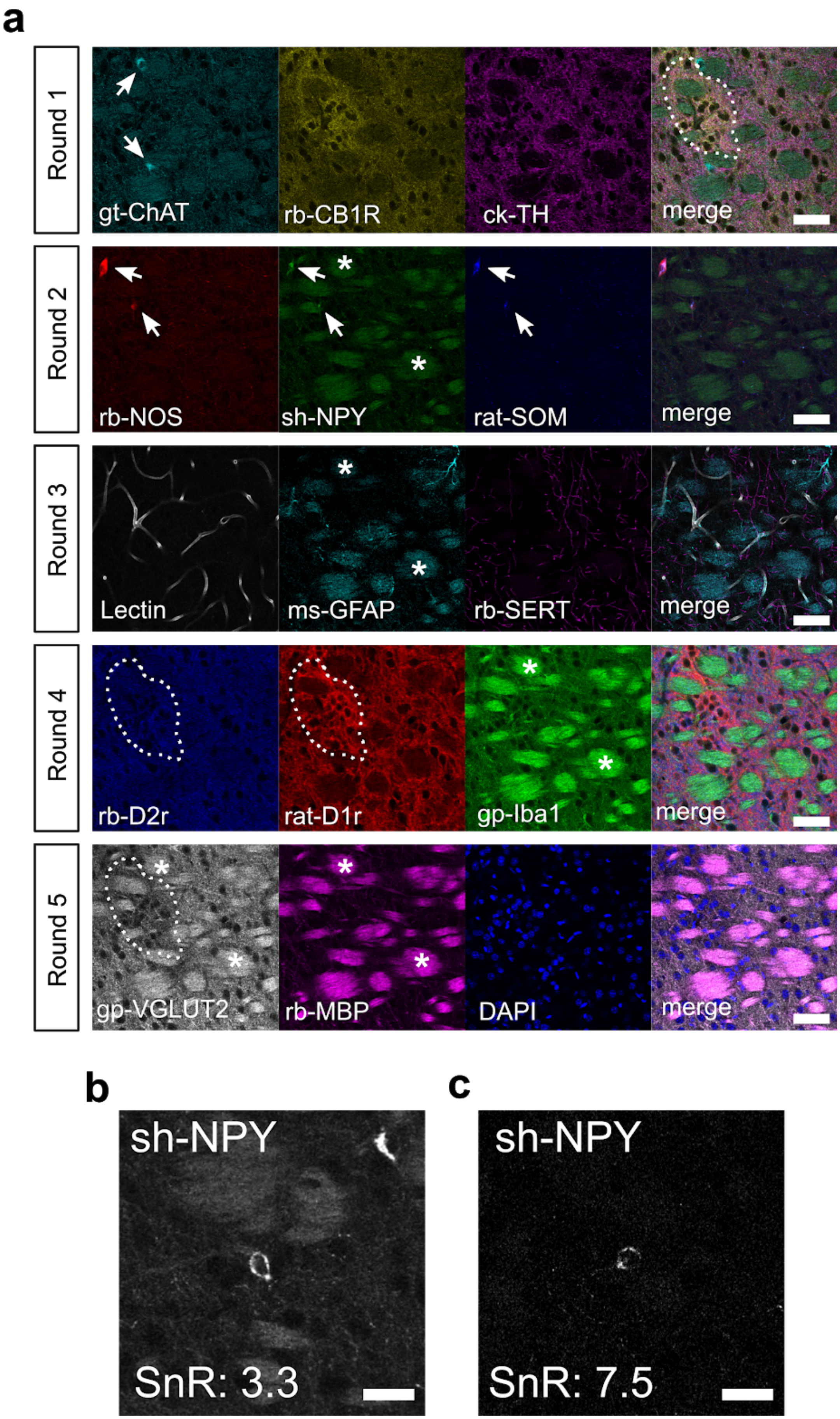
a
Round 1
gt-ChAT
rb-CB1R
ck-TH
merge
Round 2
rb-NOS
sh-NPY
rat-SOM
merge
Round 3
Lectin
ms-GFAP
rb-SERT
merge
Round 4
rb-D2r
rat-D1r
gp-Iba1
merge
Round 5
gp-VGLUT2
rb-MBP
DAPI
merge
b
sh-NPY
SnR: 3.3
c
sh-NPY
SnR: 7.5

**Supplementary Figure 5. miriEx enables highly multiplexed molecular profiling.** (a) A total of 15 different targets across 5 rounds were probed and imaged with confocal microscopy. DAPI was stained in each round to use as a fiduciary channel for registration. CB1R expression marks patches, a histochemically distinct compartment within the striatum (dashed white outline, round 1). NOS, NPY, and SOM expression largely overlap (round 2). D1r expression is higher in striatal patches, while D2r and VGLUT2 expression is higher in the surrounding matrix compartment (round 4). White arrows point to cell bodies with positive molecular marker expression. Asterisks point to myelin bundles immunostained by myelin binding protein (MBP, round 5), typically seen in coronal striatal sections. However, myelin bundles can also be non-specifically stained by secondary antibodies as high background in other rounds. (b) An example of NPY immunostaining with high background in striatum. (c) Demonstration that lowering the secondary antibody concentration two fold from 1:500 to 1:1000 decreases background and increases signal to noise (SnR). ChAT, choline acetyltransferase; CB1R, cannabinoid receptor type 1; TH, tyrosine hydroxylase; NOS, nitric oxide synthase; NPY, neuropeptide Y; SOM, somatostatin; GFAP, glial fibrillary acidic protein; SERT, serotonin transporter; D1r, dopamine receptor D1; D2r, dopamine receptor D2; Iba1, ionized calcium binding adaptor molecule 1; VGLUT2, vesicular glutamate transporter 2; MBP, myelin basic protein;  Scale bars: (a) 50 μm (pre-expansion size). (b-c) 25 μm (pre-expansion size). Expansion factor: (a-c) ~2x. See Supplementary Table 1 for more details.

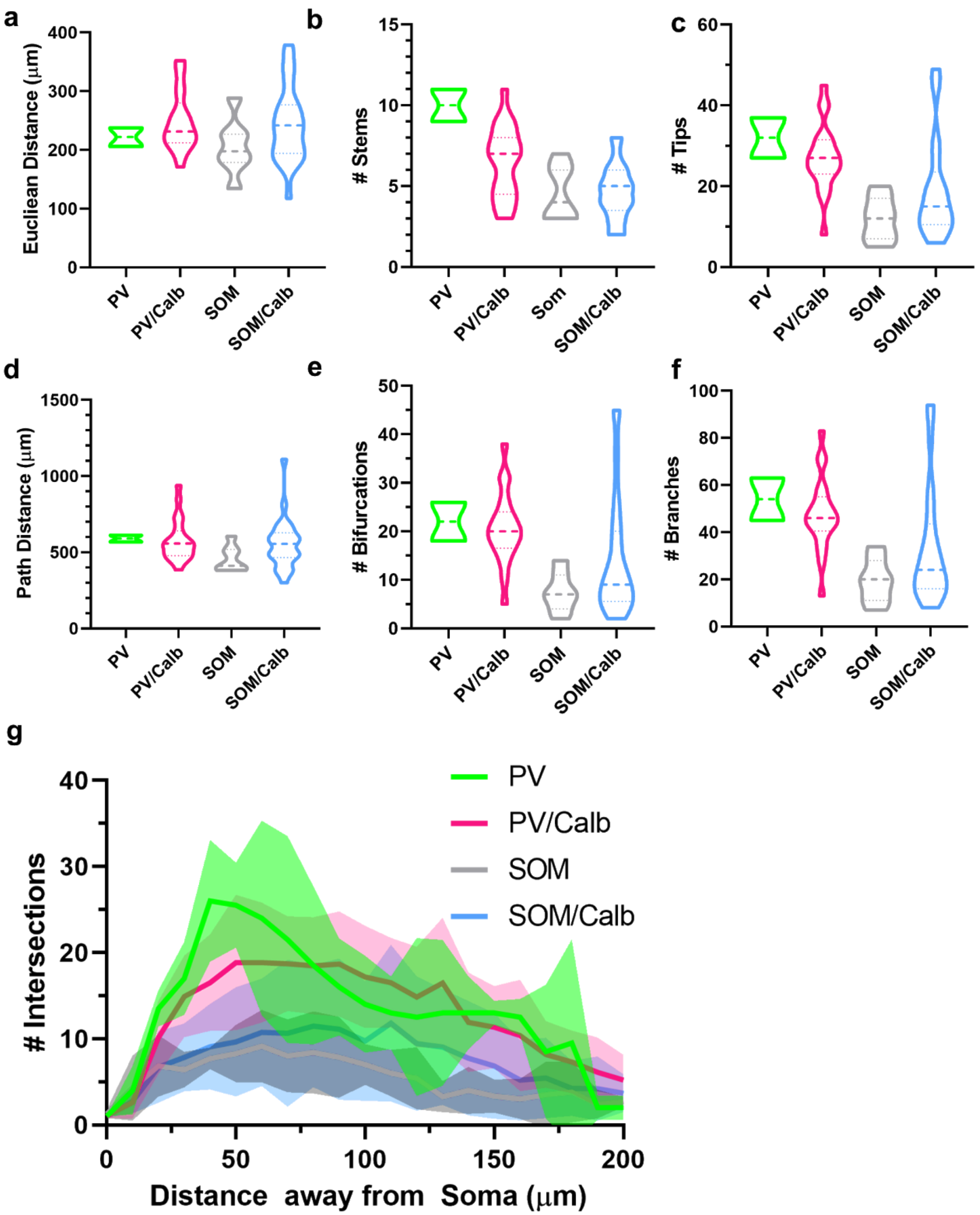


**Supplementary Figure 6. Morphology quantification of 53 inhibitory neurons traced in the BLA.** (a-f) Various morphometric parameters were calculated using Vaa3D global neuron features for each of the 4 molecular subtypes. Bolded dash lines represent the median; thin dashed lines represent quartiles. (g) 3D-Sholl analysis for each of the 4 molecular subtypes. Shaded region represents standard deviation error.

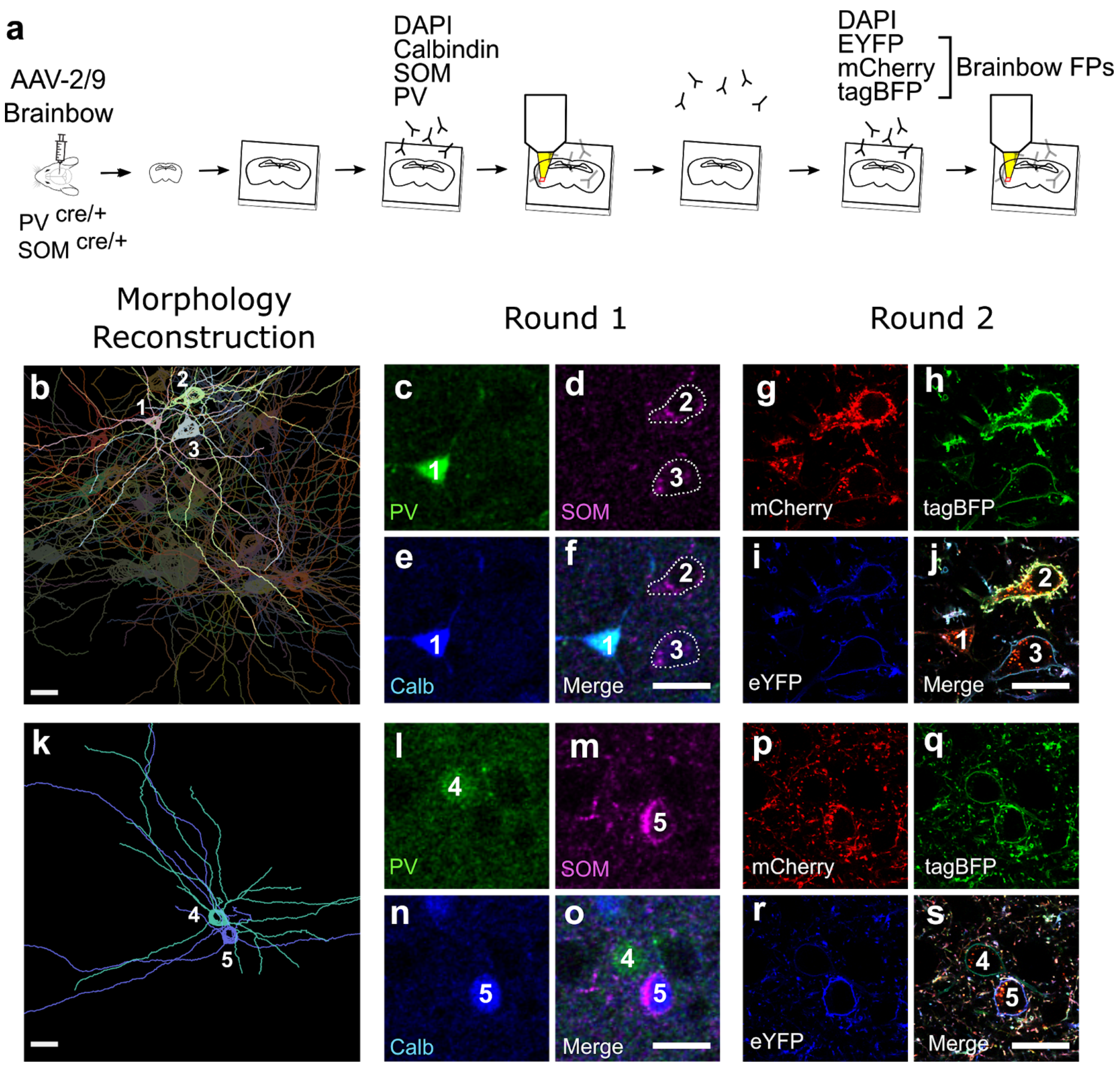


**Supplementary Figure 7. PV, PV/Calb, SOM, and SOM/Calb neuron subtype dendritic morphology can be identified in dorsal endopiriform nucleus.** (a) Experiment design: Brainbow and molecular markers were imaged across two rounds of immunostaining using the DAPI channel for registration. Both rounds of imaging took place with the gel-tissue hybrid expanded ~2x in 1xPBS, giving us an effective imaging resolution of ~150 x 150 x 350 $nm^3$. The total imaging volume was 590 x 590 x 130 $\mu m^3$. (b) Dendritic morphology reconstructions of 30 cells with somas in a subvolume (200 x 200 x 130 $\mu m^3$) of the total dataset. Associated molecular markers were identified for neurons 1-3 to show an example of PV/Calb and SOM neuron subtypes. (c-f) Single confocal slice showing the molecular markers (PV, SOM, Calb) imaged in round 1. Neuron 1 is PV and Calb double positive, while neurons 2-3 are SOM positive. (g-j) Single confocal slice showing the Brainbow channels imaged in round 2. (k) Dendritic morphology reconstruction of neurons 4-5 from a different location in the dataset than b to show an example of PV and SOM/Calb neuron subtypes. (l-o) Single confocal slice showing the molecular markers (PV, SOM, Calb) imaged in round 1. Neuron 4 is PV positive, while neuron 5 is SOM and Calb double positive. (p-s) Single confocal slice showing the Brainbow channels imaged in round 2. PV, parvalbumin; Calb, calbindin; SOM, somatostatin. Scale bars: (b-s) 20 μm (pre-expansion size). Expansion factor: (b-s) ~2x. See Supplementary Table 1 for more details.

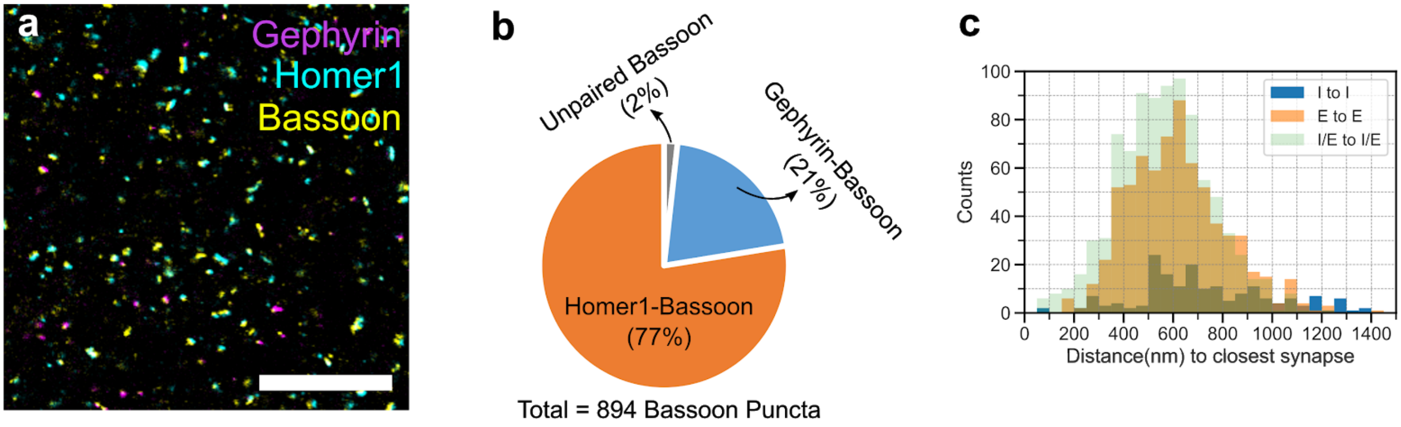


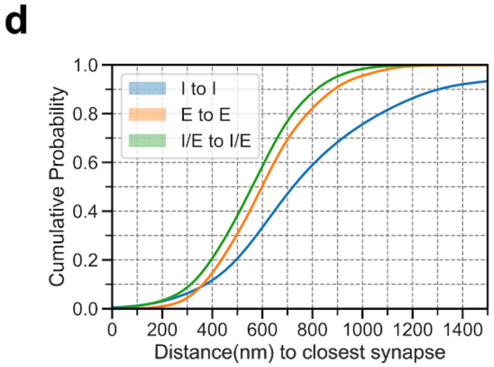


**e**

| | |
|---|---|
| Gephyrin-Bassoon Pairs/Total Gephyrin Puncta | 1 |
| Homer-Bassoon Pairs/Total Homer Puncta | 0.99 |
| Paired Bassoon/Total Bassoon Puncta | 0.98 |
| Unpaired Bassoon/Total Bassoon Puncta | 0.02 |
| Gephyrin-Bassoon Pairs/Total Bassoon Puncta | 0.21 |
| Homer-Bassoon Pairs/Total Bassoon Puncta | 0.77 |
| Gephyrin-Bassoon Density | 0.27/$\mu m^3$ |
| Homer-Bassoon Density | 1.01/$\mu m^3$ |
| Total Synapse Density | 1.28/$\mu m^3$ |

**Supplementary Figure 8. Homer1 and Gephyrin can be paired with Bassoon immunostaining to represent the majority of excitatory and inhibitory synapses, respectively.** (a) 100 μm somatosensory cortex tissue was processed with miriEx and triple immunostainied for Gephyrin, Homer1, and Bassoon. The sample was expanded ~4x and imaged. A representative slice is shown here. (b) All the synaptic pairs were manually annotated within a 15 x 15 x 3 $\mu m^3$ volume, yielding 706 excitatory synapses (Homer1-Bassoon) and 188 inhibitory synapses (Gephyrin-Bassoon). Pie chart shows that the majority of Bassoon puncta are paired with either Gephyrin or Homer1 in a mutually exclusive manner. (c) The distance between inhibitory (I) and excitatory (E) synapses to their nearest neighbor is plotted. (d) Cumulative probability plot of the histogram shown in c. (e) Table of other ratio and density measurements. Scale bars: 5 μm (pre-expansion size). Expansion factor: (a) ~4x. See Supplementary Table 1 for more details.

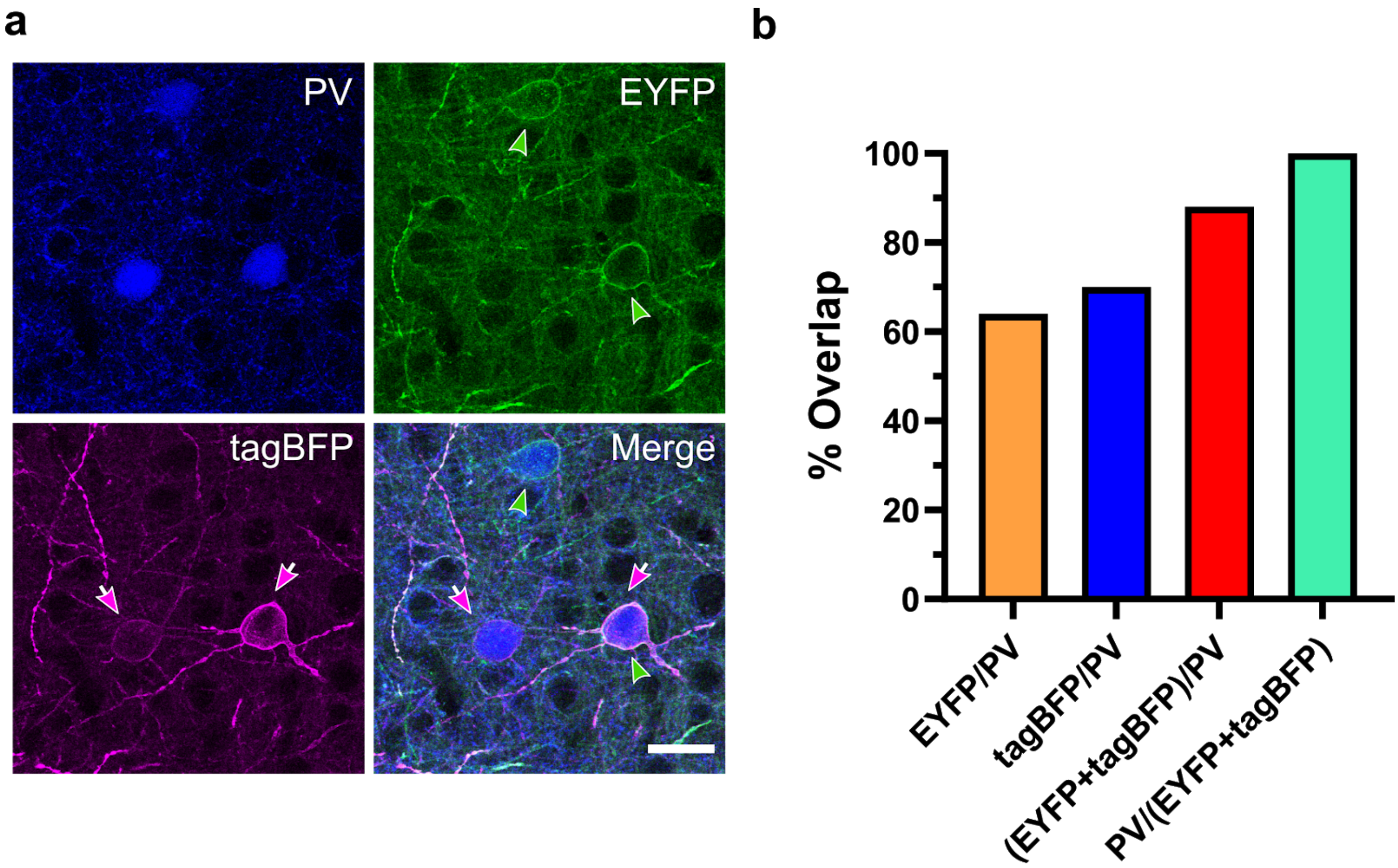


**Supplementary Figure 9. AAV-PHP.eB Brainbow expression in PV-Cre mice is highly sensitive and specific.** (a) 100 μm somatosensory cortex tissue was processed with miriEx and triple immunostainied for PV, EYFP, and tagBFP. A representative slice is shown here. (b) PV neurons across all 6 layers of somatosensory cortex were analyzed for co-expression of PV and 2 out of 4 Brainbow FPs. 88% of all PV neurons (n=50, identified through immunostaining) were positive for either EYFP or tagBFP. 100% of all Brainbow labeled neurons (n=44) were positive for PV immunostaining. PV, parvalbumin; FP, fluorescent protein. Scale bars: 50 μm (pre-expansion size). See Supplementary Table 1 for more details.

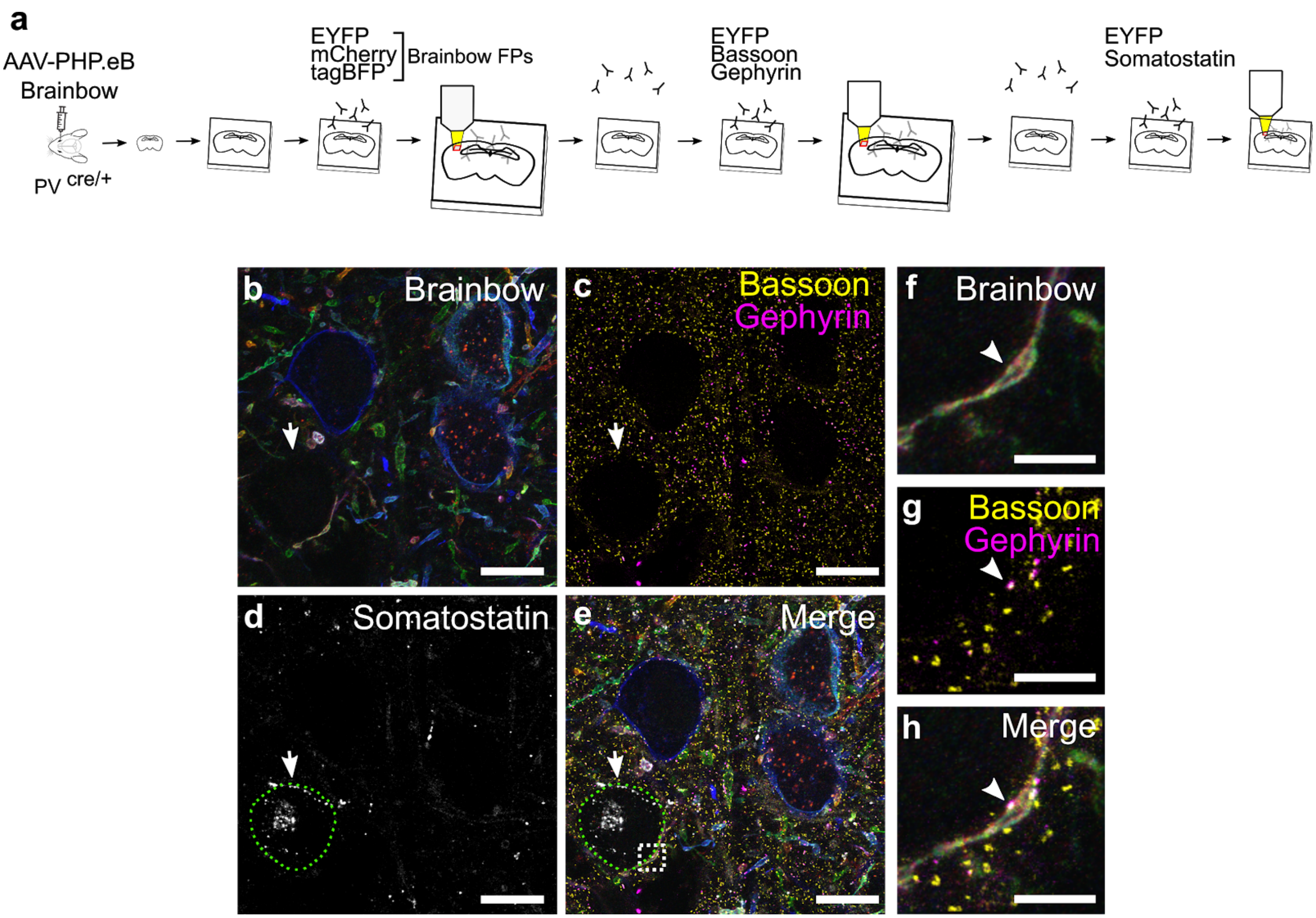


**Supplementary Figure 10. Brainbow FPs, synaptic machinery, and molecular markers can be interrogated in the same piece of tissue.** (a) Experimental design: Brainbow FPs, endogenous synaptic markers (Bassoon, Gephyrin), and cell type markers (SOM) are imaged across three rounds of immunostaining using the EYFP channel for registration. (b) ~750 nm MIP showing the Brainbow channels imaged in r1. (c) ~750 nm MIP showing the Bassoon and Gephyrin puncta imaged in r2. (d) ~750 nm MIP showing a Somatostatin positive soma. (e) Merged MIP of all three rounds. White arrow points to an "empty hole" that is later defined as a SOM positive soma outlined in a dashed green circle. (f-h) Single slice zoomed inset of white square shown in e; the arrow points to a putative synaptic connection between the PV axon and soma of the SOM neuron. FP, fluorescent protein; MIP, maximum intensity projection; SOM, somatostatin. Scale bars: (b-e) 10 μm (pre-expansion size). (f-h) 3 μm (pre-expansion size). Expansion factor: (b-c) ~4x. (d) ~2x. See Table S1 for more details.

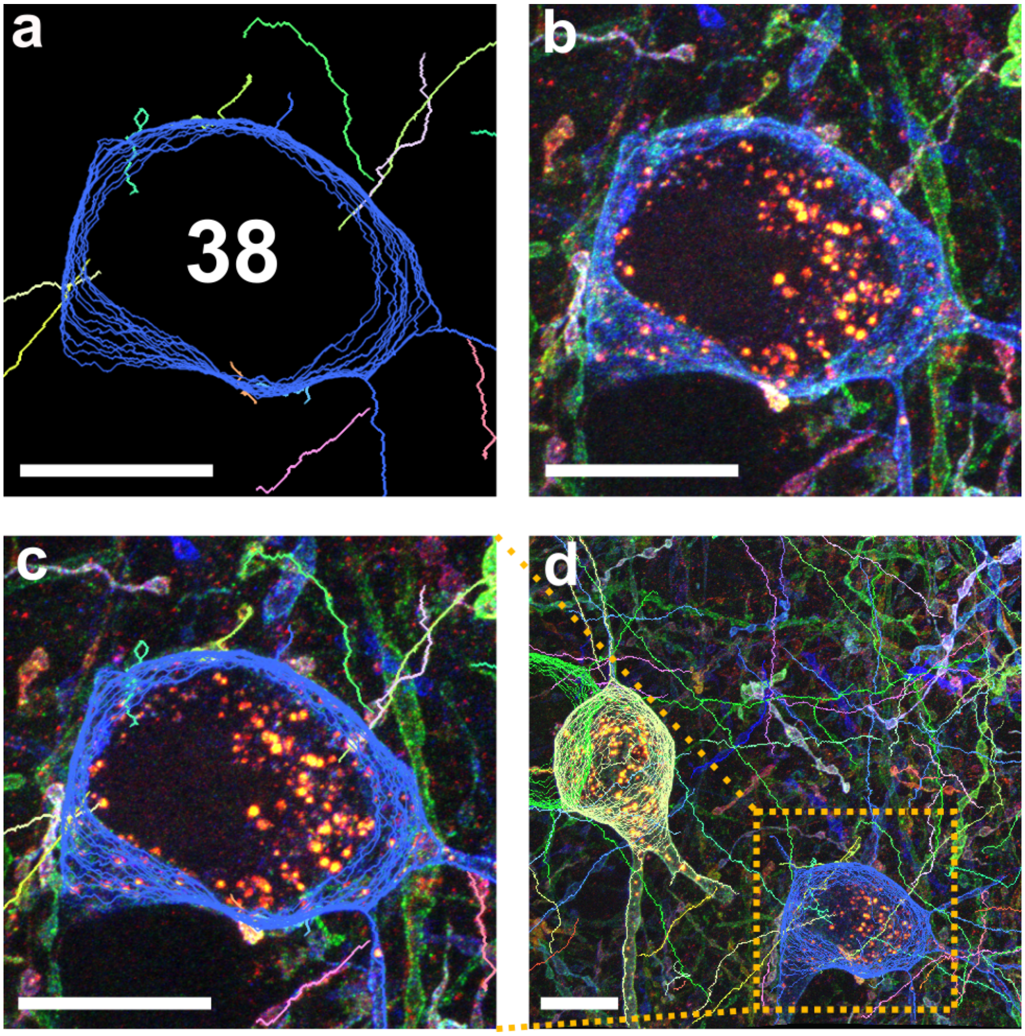


**Supplementary Figure 11. nTracer reconstructions are reliable and precise for spectral connectomic datasets.** (a) MIP nTracer reconstructions of neuron 38 and all the putatively connected local axons from Fig. 4. (b) MIP showing the composite Brainbow channels. (c) Overlay of nTracer reconstruction on the Brainbow imaging data demonstrates tracing precision and reliability. (d) Larger FOV of the overlaid image with the orange square representing b-c. The goal of the tracing from this image is to analyze the inhibitory inputs to selected PV-neurons with their somas located within the imaging volume. To accelerate the tracing, we first identified the Bassoon-Gephyrin-Brainbow trio signals on the target PV-neurons followed by tracing all the input axons "outward". We ignored the axons passing by that did not make connections with the target neurons, although they could be as confidently traced based on their similar labeling quality. MIP, maximum intensity projection. Scale bars: 10 µm (pre-expansion size). Expansion factor: (b-d) ~4x. See Supplementary Table 1 for more details.

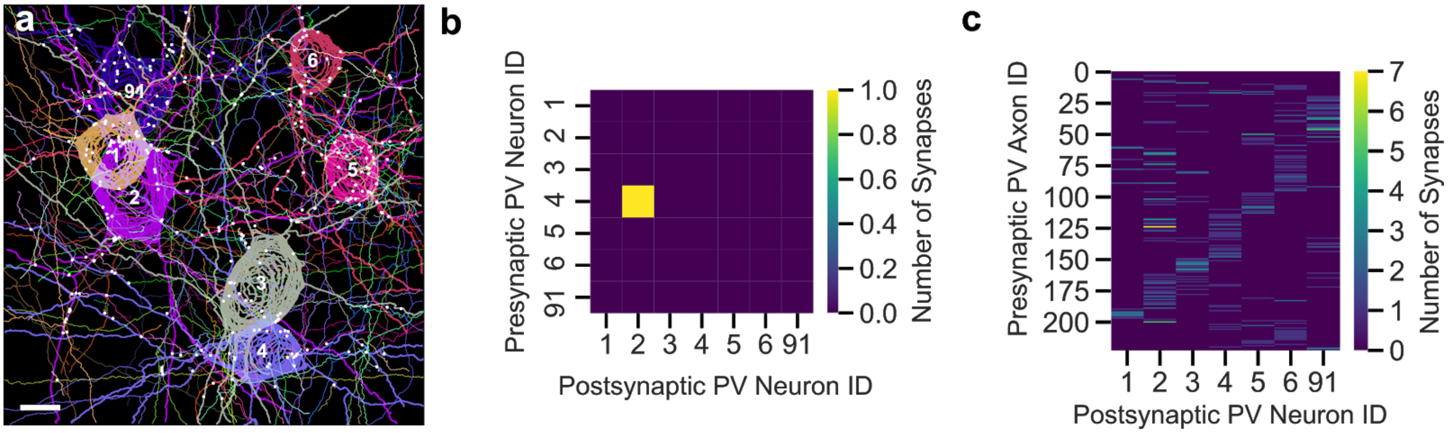


**Supplementary Figure 12. Technical repeat analyzing PV to PV connectivity.** (a) The experimental protocol from Fig. 3a was repeated on a brain section from another PV-Cre mouse injected with AAV-PHP.eB-Brainbow. MIP of 7 Brainbow labeled PV neurons reconstructed with nTracer, plus 222 innervating PV axons. Thick neurites represent dendrites; thin neurites represent axons. 332 identified synapses are marked with white circles. (b) Connectivity matrix between the 7 reconstructed PV neurons. (c) Connectivity matrix as in c, plus the 222 innervating axons. MIP, maximum intensity projection. Scale bars: 10 μm (pre-expansion size).

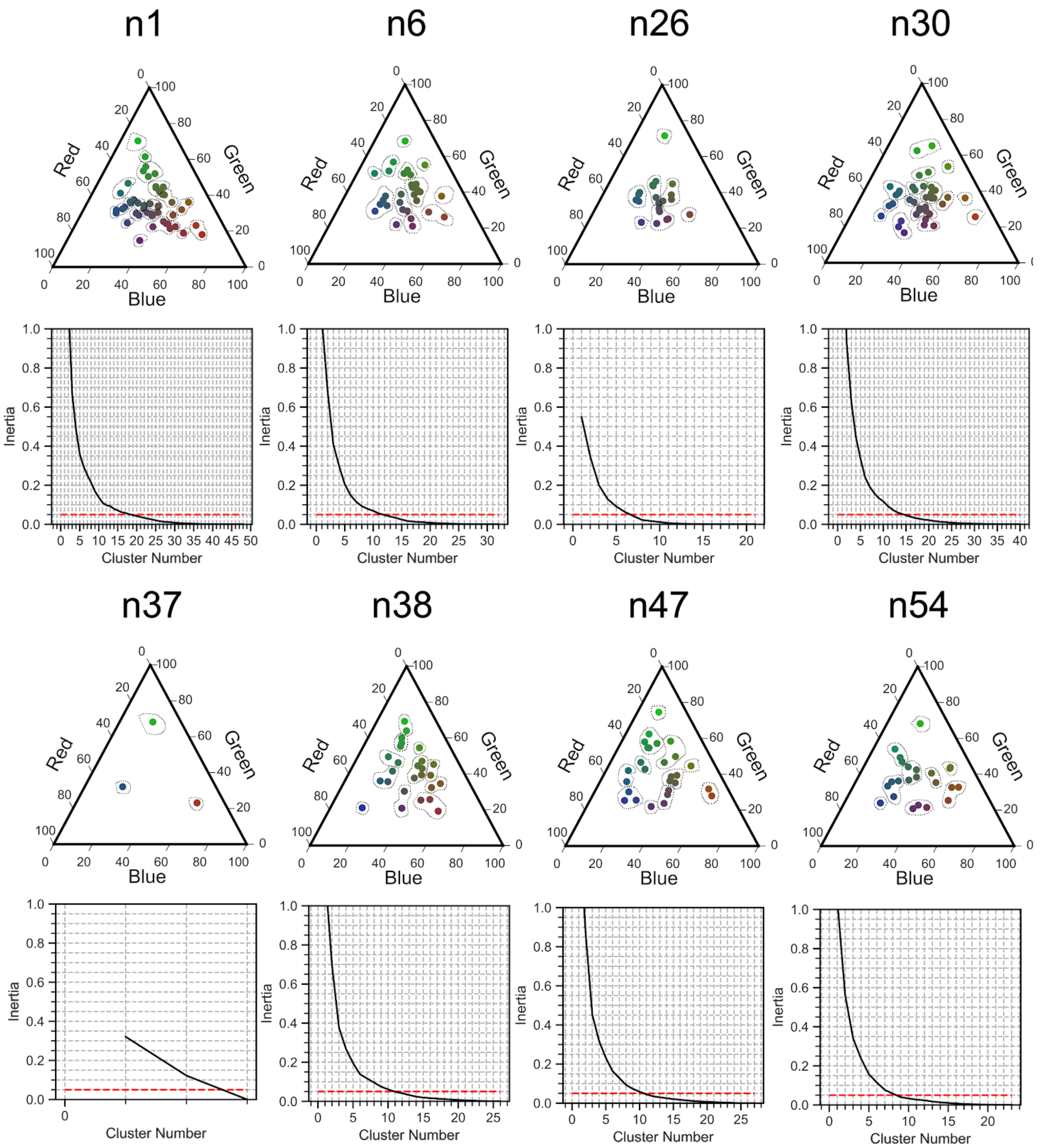


**Supplementary Figure 13. Number of unique axon colors can be identified for k-means clustering.** For each neuron, axon color identities were extracted through nTracer and plotted on a RGB color plot. An elbow plot was used to find the optimal number of k for k-means clustering. This value was conservatively chosen to be where inertia<0.05. The dashed lines represent the k-means color cluster assignments.

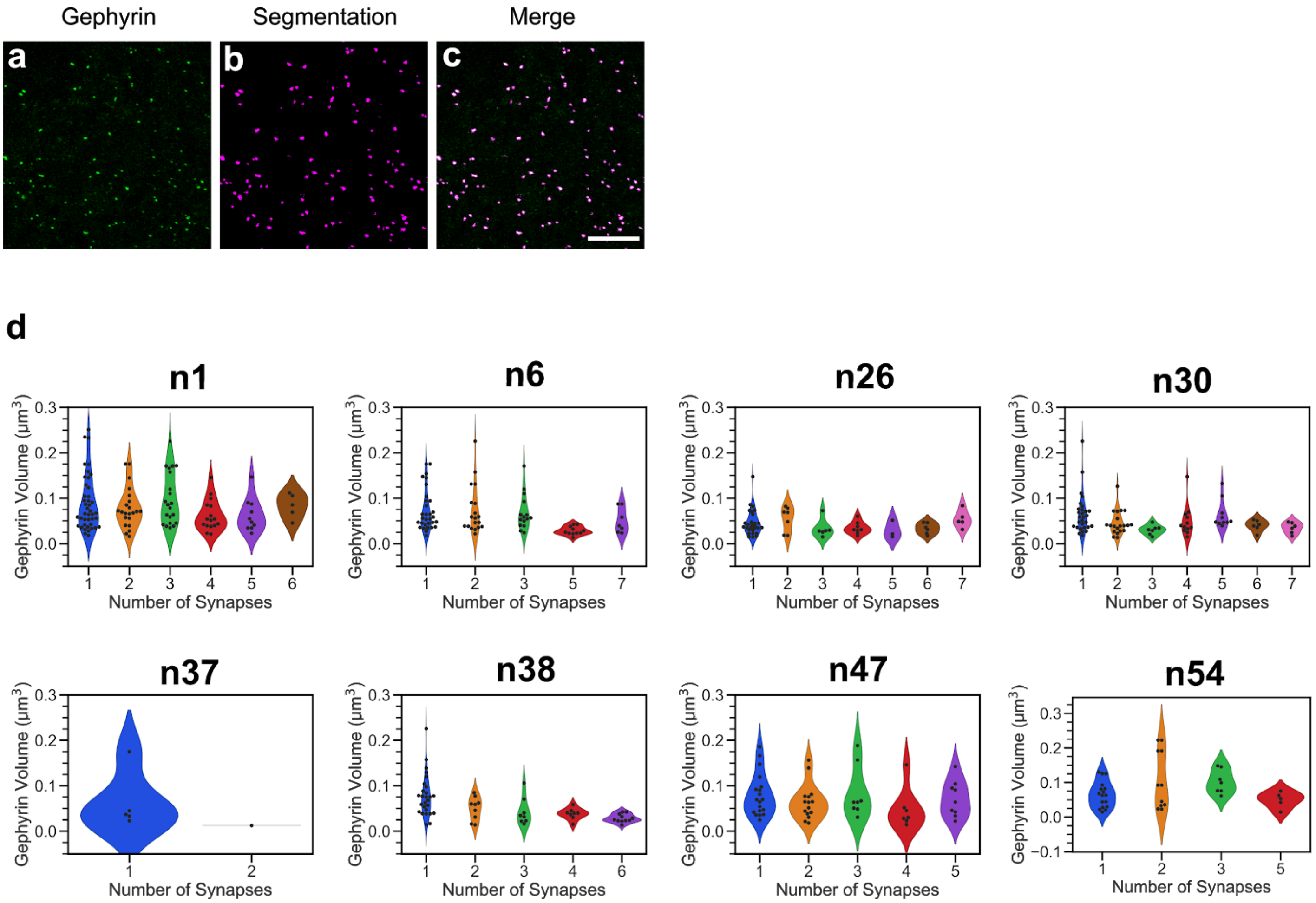


**Supplementary Figure 14. Inhibitory PSD size can be automatically segmented and analyzed.** (a) Raw Gephyrin channel. (b) Automatic segmentation result. (c) Composite image. (d) For each individual postsynaptic PV neuron, the size of the PSD was plotted as a function of the number of synapses the presynaptic PV axon formed. Scale bars: 125 nm (pre-expansion size).

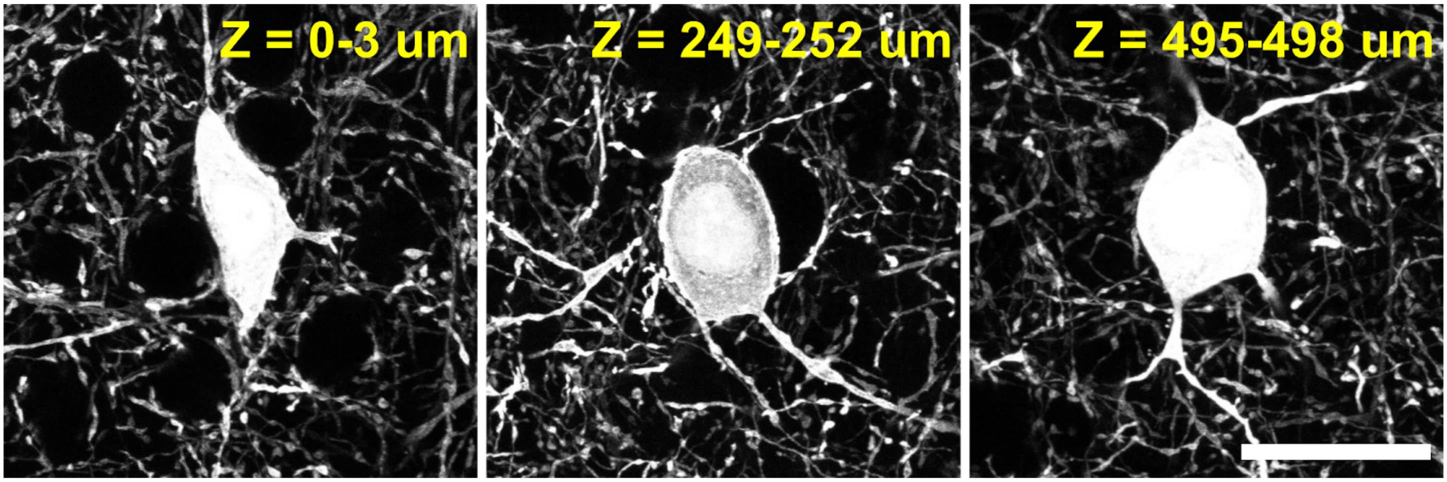


**Supplementary Figure 15. miriEx enables homogenous immunostaining in thick tissue.** 500 µm Ai14 x PV-Cre fluorescent tissue was processed with miriExl and immunostained for tdTomato. Shown are small 3 µm maximum intensity projections at the top, middle, and bottom of the tissue to demonstrate homogenous labeling. Scale bar: 25 µm (pre-expansion size). See Supplementary Table 1 for more details.

## Supplementary Table 1. Summary of experimental conditions conducted in each figure.

| Figure | Sample | [Aax] | Primary Ab | Primary incubation time and temperature | Secondary Ab | Secondary incubation time and temperature | Imaging | Processing: stitching, chromatic aberration, histogram matching |
|---|---|---|---|---|---|---|---|---|
| Fig 1 | 100 µm BLA from WT mouse | 5 mM | r1: rb-PV (1:500)<br>r2: rb-Calbindin (1:500)<br>r3: rb-CB1R (1:333)<br>r4: rb-PV (1:500)<br>r5: rb-NOS (1:500)<br>r6: rb-SERT (1:500)<br>r7: rb-PV (1:500) | r1-r7: 1d at 37c | r1-r7: rb-AF488 (1:500), DAPI | r1-r7: 1d at 37c | r1-r7: Confocal, 10x Objective (0.4 NA), imaged in 1xPBS (~2x expansion) | Yes (3x2 tiles), No, No |
| Fig 2, sFig 7 | 200 µm BLA section from PV-Cre/Som-Cre stereotaxically injected with AAV2/9 Brainbow in BLA (n=2 separate animals) | 1 mM | r1: rb-Calbindin (1:500), rat-SOMsc(1:100), gp-PV (1:500)<br>r2: rb-mCherry (1:500), sh-GFP (1:500), gp-tagBFP (1:500) | r1: 3d at RT<br>r2: 2d at 37c | r1: rat-AF488(1:500), rb-AF647 (1:500), gp-Cy3 (1:500)<br>r2: rat-AF488(1:500), rb-AF647 (1:500), gp-Cy3 (1:500) | r1: 2d at RT<br>r2: 2d at 37c | r1: Confocal, 10x objective (0.4 NA), imaged in 1x PBS (~2x expansion)<br>r2: Confocal, 20x Objective (1.0 NA), imaged in 1x PBS (~2x expansion)<br>**Fig 2b-i MIP**: 15 slices across 5.25 µm in Z | Yes (3x2 tiles), Yes, Yes |
| Fig 3-4, sFig9-12 | 100 µm S1 Cortex section from PV-Cre retroorbitally injected with PhP.eB Brainbow (n=2 separate animals) | 1 mM | r1: rb-mCherry (1:500), sh-GFP(1:500), gp-tagBFP (1:500)<br>r2: sh-GFP (1:500), gp-Bassoon (1:500), ms-GephyrinSC (1:100)<br>r3: rat-SOMsc (1:100), sh-GFP (1:500) | r1: 2d at RT<br>r2: 2d at 37c<br>r3: 2d at 37c | r1: rb-AF488 (1:500), sh-Cy3 (1:500), gp-AF647 (1:500)<br>r2: ms-AF488 (1:500), sh-Cy3 (1:500), gp-AF647 (1:500)<br>r3: sh-Cy3 (1:500), rat-AF647 (1:500) | r1: 1d at RT<br>r2: 1d at 37c<br>r3: 1d at 37c | r1: confocal, 20x objective (1.0 NA), imaged in 0.001x PBS (~4x expansion)<br>r2: confocal, 20x objective (1.0 NA), imaged in 0.001x PBS (~4x expansion)<br>r3: confocal, 20x objective (1.0 NA), imaged in 1x PBS (~2x expansion)<br>**Fig 3b-c MIP**: 40 slices across 6.0 µm in Z<br>**sFig 10b-d:** 5 slices across 750 nm in Z<br>**sFig 11a-c MIP**: 40 slices across 6.0 µm in Z<br>**sFig 12a MIP:** 511 slices across 66 µm in Z | No, Yes, Yes |
| Fig 5 | 100 µm BLA section from PV-Cre stereotaxically injected with AAV2/9 Brainbow in BLA (n=1 animal) | 1 mM | r1: rb-mCherry (1:500), sh-GFP (1:500), gp-tagBFP (1:500)<br>r2: gp-Bassoon (1:500), ms-GephyrinSC (1:100), sh-GFP (1:500) | r1: 2d at 37c<br>r2: 1d at 37c | r1: gp-AF647 (1:500), ms-AF488 (1:500), sh-Cy3 (1:500)<br>r2: rb-AF488 (1:500), gp-AF647 (1:500), sh-Cy3 (1:500) | r1-r2: 1d at 37c | r1: Confocal, 20x Objective (1.0 NA), imaged in 0.001x PBS (~4x expansion)<br>r2: Confocal, 20x Objective (1.0 NA), imaged in 0.001x PBS (~4x expansion)<br>**Fig 5b MIP:** 55 slices across 9.635 µm in Z | Yes (2x2 tile), Yes, Yes |
| sFig 1 | 100 µm VGAT-Cre x Ai14 S1 cortex | 1 mM | r1: rb-mCherry (1:500) | r1: 1d at 37c | r1: rb-AF488 (1:500)<br>post strip: rb-AF647 (1:500) | r1/post strip: 1d at 37c | Confocal, 10x objective(0.4 NA) , imaged in 1x PBS (~2x expansion) | No, No, No |
| sFig 2 | 100 µm Thy1-YFP-H S1 cortex | 1 mM | sh-GFP (1:500) | 1 day at 37c | sh-AF594 (1:500) | 1d at 37c | Confocal, 10x Objective (0.4 NA), imaged in Vectashield, 1xPBS (~2x expansion),0.001x PBS (~4x expansion) | No, No, No |
| sFig 4 | 100 µm human sensory cortex | 5 mM | r1: rb-Calbindin (1:500), gp-Calretinin (1:500), ms-GFAP (1:500) r2: ms-SMI312 (1:333), rb-PV(1:500) | r1-r2: 1d at 37c | r1: rb-Cy3 (1:500), gp-AF488 (1:500), ms-AF647 (1:500), DAPI<br>r2: ms-AF488 (1:500), rb-AF647 (1:500), DAPI | r1-r2: 1d at 37c | r1-r2: Confocal, 10x objective (0.4 NA), imaged in 1xPBS (~2x expansion) | Yes (2x1 tile), No, No |
| sFig 5 | 100 µm WT dorsolateral striatum | 5mM | r1: gt-ChAT (1:500), rb-CB1R(1:500), ck-TH (1:500)<br>r2: rb-NOS (1:500), sh-NPY(1:500), rat-SOM (1:100)<br>r3: Lectin (1:1000), ms-GFAP (1:500), rb-SERT (1:500) r4: rb-D2R (1:500), rat-D1R(1:500), gp-Iba1(1:300) r5: gp-VGLUT2 (1:500), rb-MBP (1:500) | r1-r5: 1d at 37c | r1: ck-AF488 (1:500), rb-Cy3 (1:500), gt-CF647 (1:500), DAPI<br>r2: sh-AF488 (1:500), rb-Cy3 (1:500), rat-AF647 (1:500), DAPI<br>r3: ms-AF488 (1:500), rb-AF647 (1:500), DAPI<br>r4: gp-AF488 (1:500), rb-Cy3 (1:500), rat-AF647 (1:500), DAPI<br>r5: gp-AF488 (1:500), rb-Cy3 (1:500) | r1-r5: 1d at 37c | r1-r5: Confocal, 10x objective (NA 0.4), imaged in 1x PBS (~2x expansion) | Yes (2x2 tile), No, No |
| sFig 8 | 100 µm S1 cortex from WT mouse | 1 mM | rb-Homer1a (1:500), ms-GephyrinSC (1:100), gp-Bassoon (1:500) | 2d at 37c | ms-AF488 (1:500), rb-Cy3 (1:500), gp-AF647 (1:500) | 1d at 37c | Confocal, 20x objective (1.0 NA), imaged in 0.001x PBS (~4x expansion) | No, Yes, Yes |
| sFig 9 | 100 µm S1 Cortex section from PV-Cre retroorbitally injected with PhP.eB Brainbow (same brain as Fig.3) | 1 mM | gp-tagBFP(1:500), rb-PV (1:500), sh-GFP (1:500) | 1d at 37c | gp-AF647 (1:500), sh-Cy3 (1:500), rb-AF488 (1:500) | 1d at 37c | Confocal, 10x objective (0.4 NA), imaged in 1xPBS (~2x expansion) | Yes (2x2 tile), Yes, Yes |
| sFig 15 | 500 µm PV-Cre x Ai14 S1 cortex | 1mM | rb-mCherry (1:500) | 5d at 37c | rb-AF488 (1:500) | 3d at 37c | 20x Objective (1.0 NA), imaged in 1x PBS (~2x expansion)<br>**sFig 15 MIP**: 10 slices across 3 µm in Z | No, No, No |

**Supplementary Table 2. List of primary antibodies used in this study.**

| Target | Vendor | Catalog Number | Host Species |
|---|---|---|---|
| VGAT | Synaptic Systems | 131 011 | Ms |
| Calbindin | Synaptic Systems | 214 002 | Rb |
| PV | Synaptic Systems | 195 004 | Gp |
| PV | Abcam | ab32895 | Gt |
| Homer1 | Synaptic Systems | 160 002 | Rb |
| Gephyrin | Synaptic Systems | 147 111 | Ms |
| Bassoon | Synaptic Systems | 141 004 | Gp |
| CamKII | Abcam | ab22609 | Ms |
| TH | Abcam | ab76442 | Ck |
| NOS | Sigma | n2780 | Rb |
| NPY | Millipore | ab1583 | Sh |
| Somatostatin | Millipore | mab354 | Rt |
| VIP | Immunostar | 20077 | Rb |
| Calretinin | Synaptic Systems | 214 104 | Gp |
| SMI-312 | Biolegend | 837904 | Ms |
| SERT | Synaptic Systems | 340 003 | Rb |
| CB1R | Synaptic Systems | 258 003 | Rb |
| D2R | Synaptic Systems | 376 203 | Rb |
| D1R | Sigma | D2944 | Rt |
| GFAP | Dako Agilent | Z033401 | Rb |
| Vglut2 | Synaptic Systems | 135 404 | Gp |
| IBA1 | Synaptic Systems | 234 004 | Gp |
| MBP | Synaptic Systems | 295 002 | Rb |
| Lectin | Vector Labs | DL-1177 | N/A |
| GAD67 | Millipore | mab5406 | Ms |
| SomatostatinSC | Santa Cruz | YC7 | Rt |
| GephyrinSC | Santa Cruz | G6 | Ms |
| PV | Abcam | ab11427 | Rb |
| NeuN | Millipore | mab377 | Ms |
| Homer1 | Synaptic Systems | 160 006 | Ck |
| GFAP | Sigma | G3893 | Ms |
| CTIP2 | Abcam | ab18465 | Rt |
| tagBFP | Cai Lab custom made | NA | Gp |
| mCherry | Cai Lab custom made | NA | Rb |
| GFP | Biorad | 4745-1051 | Sh |

Rb: Rabbit; Gp: Guinea pig; Sh: Sheep; Rt: Rat; Ck: Chick; Ms: Mouse; Gt: Goat; N/A: Not Applicable

**Supplementary Table 3. List of fluorescent dye conjugated secondary antibodies used in this study.**

| Target | Vendor | Catalog Number | Host Species |
|---|---|---|---|
| Rb-AF488 | Jackson Immunoresearch | 711-545-152 | Dk |
| Rb-Cy3 | Jackson Immunoresearch | 711-166-152 | Dk |
| Rb-AF647 | Jackson Immunoresearch | 711-605-152 | Dk |
| Gp-AF488 | Jackson Immunoresearch | 706-545-148 | Dk |
| Gp-Cy3 | Jackson Immunoresearch | 706-166-148 | Dk |
| Gp-AF647 | Jackson Immunoresearch | 706-606-148 | Dk |
| Sh-AF488 | Jackson Immunoresearch | 713-546-147 | Dk |
| Sh-Cy3 | Jackson Immunoresearch | 713-166-147 | Dk |
| Rt-AF488 | Jackson Immunoresearch | 712-545-150 | Dk |
| Rt-AF647 | Jackson Immunoresearch | 712-606-153 | Dk |
| Ck-AF488 | Jackson Immunoresearch | 703-546-155 | Dk |
| Ck-AF647 | Jackson Immunoresearch | 703-606-155 | Dk |
| Ms-AF488 | Jackson Immunoresearch | 715-546-151 | Dk |
| Ms-Cy3 | Jackson Immunoresearch | 715-166-151 | Dk |
| Ms-AF647 | Jackson Immunoresearch | 715-606-151 | Dk |

Rb: Rabbit; Gp: Guinea pig; Sh: Sheep; Rt: Rat; Ck: Chick; Ms: Mouse
