# Supplementary material for: Light microscopy based approach for mapping connectivity with molecular specificity
Source: Nat Commun. 2020 Sep 15;11:4632. doi: 10.1038/s41467-020-18422-8 (PMC7493953; doi:10.1038/s41467-020-18422-8)
Supplement: Supplementary file 3 — Description of Additional Supplementary Files [file 41467_2020_18422_MOESM3_ESM.pdf]

Description of Additional Supplementary Files

File Name: Supplementary Movie 1
Description: **Dendritic morphology reconstructions of molecularly defined inhibitory neuronal subtypes.** Related to Figure 2. Inhibitory neurons in the basal lateral amygdala (BLA) were labeled by Brainbow AAVs. Expression of parvalbumin (PV), somatostatin (SOM), or calbindin (Calb) along with Brainbow labeling can be specified by miriEx. nTracer reconstructions of the dendritic morphology of each molecular neuronal subtype are shown.

File Name: Supplementary Movie 2
Description: **Spectral connectomics reveals inhibitory connections between molecularly defined neurons in the mouse brain.** Related to Figure 3. Mouse cortical parvalbumin (PV) expressing neurons are labeled by Brainbow. MiriEx processing conformed PV expression, visualized Brianbow labeling, and localized pre- and post-inhibitory synapse markers in multiple rounds of imaging. Arrowheads indicate inhibitory synaptic contacts being identified by spatial correlation of the Bassoon-Brainbow-Gephyrin trio signals.

File Name: Supplementary Movie 3
Description: **3D rendering of a putative inhibitory synapse formed between two parvalbumin (PV) expressing neurons labeled in distinct colors.** Related to Figure 3. Blue: Soma of the post-synaptic PV neuron. Orange: Axonal bouton of the pre-synaptic PV neuron. Green: Gephyrin signal marks the post-synaptic densities. Red: Bassoon signal marks the pre-synaptic densities. Yellow: Convoluted pre- and post-synaptic signals that cannot be resolved by light microscopy, especially in the axial direction.

File Name: Supplementary Movie 4
Description: **nTracer reconstructed neuron morphology and putative inhibitory synapses of parvalbumin (PV) expression neurons.** Related to Figure 4. 8 PV neurons with somas and the PV axon that innervated these 8 neurons were traced by nTracer. All the putative inhibitory synapses between these neurons were annotated in white dots.

File Name: Supplementary Movie 5
Description: **3D visualization of putative excitatory and inhibitory synapses identified on reconstructed aspiny inhibitory neurons by nTracer.** Related to Figure 5. Parvalbumin (PV) expressing neurons are labeled by Brainbow and their soma and dendrites are reconstructed using nTracer. Endogenous post-synaptic density markers Homer1 and Gephyrin were stained and those allocated on the PV neurons were annotated as putative excitatory and inhibitory synaptic inputs, respectively.
