# Supplementary material for: Light microscopy based approach for mapping connectivity with molecular specificity
Source: Nat Commun. 2020 Sep 15;11:4632. doi: 10.1038/s41467-020-18422-8 (PMC7493953; doi:10.1038/s41467-020-18422-8)
Supplement: Supplementary file 9 — Reporting Summary [file 41467_2020_18422_MOESM9_ESM.pdf]

nature research

Corresponding author(s): Dawen Cai

Last updated by author(s): Aug 10, 2020

Reporting Summary

Nature Research wishes to improve the reproducibility of the work that we publish. This form provides structure for consistency and transparency in reporting. For further information on Nature Research policies, see our Editorial Policies and the Editorial Policy Checklist.

Statistics

For all statistical analyses, confirm that the following items are present in the figure legend, table legend, main text, or Methods section.

n/a	Confirmed	
☐	☒	The exact sample size (*n*) for each experimental group/condition, given as a discrete number and unit of measurement
☐	☒	A statement on whether measurements were taken from distinct samples or whether the same sample was measured repeatedly
☒	☐	The statistical test(s) used AND whether they are one- or two-sided<br>*Only common tests should be described solely by name; describe more complex techniques in the Methods section.*
☒	☐	A description of all covariates tested
☒	☐	A description of any assumptions or corrections, such as tests of normality and adjustment for multiple comparisons
☐	☒	A full description of the statistical parameters including central tendency (e.g. means) or other basic estimates (e.g. regression coefficient) AND variation (e.g. standard deviation) or associated estimates of uncertainty (e.g. confidence intervals)
☒	☐	For null hypothesis testing, the test statistic (e.g. *F*, *t*, *r*) with confidence intervals, effect sizes, degrees of freedom and *P* value noted<br>*Give P values as exact values whenever suitable.*
☒	☐	For Bayesian analysis, information on the choice of priors and Markov chain Monte Carlo settings
☒	☐	For hierarchical and complex designs, identification of the appropriate level for tests and full reporting of outcomes
☒	☐	Estimates of effect sizes (e.g. Cohen's *d*, Pearson's *r*), indicating how they were calculated

Our web collection on statistics for biologists contains articles on many of the points above.

Software and code

Policy information about availability of computer code

Data collection	All imaging was done on an upright Zeiss LSM780 confocal microscope.
Data analysis	Histogram matching, and Brainbow neuron reconstruction was performed using Fiji/ImageJ plugin nTracer-1.3.5.<br>Chromatic aberration was corrected using Fiji/ImageJ plugin Detection of Molecules-1.1.6.<br>Multi-tile stitching was performed using Fiji/ImageJ plugin BigStitcher-0.3.3.<br>Putative Synapses were annotated using ROI manager in Fiji/ImageJ 1.50e.<br>Elastix v5.0 was used for image registration.<br>Blender v2.7 and Fiji/ImageJ plugin 3DScript was used for making movies.<br>Custom python Jupyter lab scripts are available at https://github.com/fshen11/spectral-connectomics

For manuscripts utilizing custom algorithms or software that are central to the research but not yet described in published literature, software must be made available to editors and reviewers. We strongly encourage code deposition in a community repository (e.g. GitHub). See the Nature Research guidelines for submitting code & software for further information.

Data

Policy information about availability of data

All manuscripts must include a data availability statement. This statement should provide the following information, where applicable:

- Accession codes, unique identifiers, or web links for publicly available datasets
- A list of figures that have associated raw data
- A description of any restrictions on data availability

The data that support the findings of this study are available from the corresponding author upon reasonable request.

Field-specific reporting

Please select the one below that is the best fit for your research. If you are not sure, read the appropriate sections before making your selection.

☒ Life sciences ☐ Behavioural & social sciences ☐ Ecological, evolutionary & environmental sciences

For a reference copy of the document with all sections, see nature.com/documents/nr-reporting-summary-flat.pdf

Life sciences study design

All studies must disclose on these points even when the disclosure is negative.

Sample size	As a methods paper, no sample size calculation was performed. We chose a minimum of 2 technical replicates for each experiment to demonstrate reproducibility
Data exclusions	No data was excluded from the analysis.
Replication	All antibodies used were replicated and successfully pre-tested in individual experiments to validate their efficacy and sensitivity. For the experiments related to Figures 2,3, and 4, an additional technical replicate was successfully performed once to demonstrate reproducibility. Replicates are shown in the supplemental figures.
Randomization	No randomization was performed. Randomization was not necessary as the goal of the study is to demonstrate a new technique and not make biological conclusions.
Blinding	No blinding was performed. Blinding was not necessary as the goal of the study is to demonstrate a new technique and not make biological conclusions.

Reporting for specific materials, systems and methods

We require information from authors about some types of materials, experimental systems and methods used in many studies. Here, indicate whether each material, system or method listed is relevant to your study. If you are not sure if a list item applies to your research, read the appropriate section before selecting a response.

Materials & experimental systems

n/a	Involved in the study	
☐	☒	Antibodies
☒	☐	Eukaryotic cell lines
☒	☐	Palaeontology and archaeology
☐	☒	Animals and other organisms
☒	☐	Human research participants
☒	☐	Clinical data
☒	☐	Dual use research of concern

Methods

n/a	Involved in the study	
☒	☐	ChIP-seq
☒	☐	Flow cytometry
☒	☐	MRI-based neuroimaging

Antibodies

Antibodies used

Ms VGAT Synaptic Systems 131 011
Rb Calbindin Synaptic Systems 214 002
Gp PV Synaptic Systems 195 004
Gt PV Abcam ab32895
Rb Homer1 Synaptic Systems 160 002
Ms Gephyrin Synaptic Systems 147 111
Gp Bassoon Synaptic Systems 141 004
Ms CamKII Abcam ab22609
Ck TH Abcam ab76442
Rb NOS Sigma n2780
Sh NPY Millipore ab1583
Rat Somatostatin Millipore mab354
Rb VIP Immunostar 20077
Gp Calretinin Synaptic Systems 214 104
Ms SMI-312 Biolegend 837904
Rb SERT Synaptic Systems 340 003
Rb CB1R Synaptic Systems 258 003
Rb D2R Synaptic Systems 376 203
Rat D1R Sigma D2944
Rb GFAP Dako Agilent Z033401

Gp Vglut2 Synaptic Systems 135 404
Gp IBA1 Synaptic Systems 234 004
Rb MBP Synaptic Systems 295 002
Lectin Vector Labs DL-1177
Ms GAD67 Millipore mab5406
Rat SomatostatinSC Santa Cruz YC7
Ms GephyrinSC Santa Cruz G6
Rb PV Abcam ab11427
Ms NeuN Millipore mab377
Ck Homer1 Synaptic Systems 160 006
Ms GFAP Sigma G3893
Rat CTIP2 Abcam ab18465
Gp tagBFP Cai Lab custom made NA
Rb mCherry Cai Lab custom made NA
Sh GFP Biorad 4745-1051
Dk Rb-AF488 Jackson Immunoresearch 711-545-152
Dk Rb-Cy3 Jackson Immunoresearch 711-166-152
Dk Rb-AF647 Jackson Immunoresearch 711-605-152
Dk Gp-AF488 Jackson Immunoresearch 706-545-148
Dk Gp-Cy3 Jackson Immunoresearch 706-166-148
Dk Gp-AF647 Jackson Immunoresearch 706-606-148
Dk Sh-AF488 Jackson Immunoresearch 713-546-147
Dk Sh-Cy3 Jackson Immunoresearch 713-166-147
Dk Rat-AF488 Jackson Immunoresearch 712-545-150
Dk Rat-AF647 Jackson Immunoresearch 712-606-153
Dk Ck-AF488 Jackson Immunoresearch 703-546-155
Dk Ck-AF647 Jackson Immunoresearch 703-606-155
Dk Ms-AF488 Jackson Immunoresearch 715-546-151
Dk Ms-Cy3 Jackson Immunoresearch 715-166-151
Dk Ms-AF647 Jackson Immunoresearch 715-606-151

Validation

All antibodies that were used have been validated in published studies (Ku et al., 2016, Nature Biotechnology. Tillberg et al., Nature Biotechnology. Cai et al., 2013, Nature Methods) and/or by the manufacturer. Rb-Calbindin, Gp-PV, Rb-PV, Rb-Homer1, Gp-Bassoon, Rb-NOS, Sh-NPY, Rat-SomatostatinSC, Ms-GephyrinSC, Rb-SERT, Rb-CB1R, Rb-D2R, Rat-D1R, Gp-Vglut2, Gp-IBA1, Rb-MBP, Lectin, Ms-GFAP, Gp-TagBFP, Rb-mCherry, Sh-GFP were all validated in this study, with images seen in the main figures and supplemental figures.

Animals and other organisms

Policy information about studies involving animals; ARRIVE guidelines recommended for reporting animal research

Laboratory animals

C57/BL6 mice, male and female, 3-8 weeks old. All experiments were carried out in accordance with a protocol approved by the University of Michigan Institutional Animal Care and Use Community. Mice were maintained on a standard 12 hour day:night cycle, with ad libitum access to food and water, at ambient temperature of 71F and humidity of 37-50%.

Wild animals

This study did not involve wild animals.

Field-collected samples

This study did not involve field-collected samples.

Ethics oversight

Our study protocol (PRO00009289, expiration date 10/07/2022) was reviewed and approved by the University of Michigan Institutional Animal Care & Use Committee.

Note that full information on the approval of the study protocol must also be provided in the manuscript.
